# 外泌体的核酸适配体筛选技术研究进展

**DOI:** 10.3724/SP.J.1123.2024.10029

**Published:** 2025-05-08

**Authors:** Liting ZHENG, Ge YANG, Feng QU

**Affiliations:** 1.北京理工大学生命学院, 北京 100081; 1. School of Life Science, Beijing Institute of Technology, Beijing 100081, China; 2.中国医学科学院北京协和医学院医药生物技术研究所, 北京 100050; 2. Institute of Medicinal Biotechnology, Chinese Academy of Medical Sciences and Peking Union Medical College, Beijing 100050, China

**Keywords:** 适配体, 指数富集系统进化技术, 外泌体, aptamer, systematic evolution of ligands by exponential enrichment (SELEX), exosome

## Abstract

外泌体密切参与细胞间通讯,调控细胞生理过程,是目前癌症和其他疾病早期诊断的潜在生物标志物之一。因此,具有特定功能的外泌体的检测与分离具有重要临床意义。与此同时,开发低成本、高灵敏度的外泌体特异性识别元件,将在疾病早期诊断和治疗过程中发挥关键作用。核酸适配体是通过指数富集配体系统进化技术(systematic evolution of ligands by exponential enrichment, SELEX)筛选获得的、可与靶标特异性结合的单链脱氧核糖核酸或核糖核酸,具备稳定性高、可化学合成、高亲和力和特异性以及靶标范围广等独特优势。目前,靶向外泌体的适配体已应用于细胞成像、药物递送、疾病诊疗等众多研究领域。然而,外泌体的异质性以及复杂结构,为精准识别特定外泌体的适配体筛选带来巨大挑战,高效的筛选技术是获得优良性能适配体的关键。本文总结了外泌体适配体筛选过程中关键靶点的功能及其选择策略,概述了目前应用于外泌体适配体筛选的主要方法与应用,包括磁珠-SELEX、微流控-SELEX、硝酸纤维素膜-SELEX、细胞-SELEX和毛细管电泳-SELEX等5种筛选技术,并对其分离原理、优势与限制以及最新应用进行阐述与分析。最后,对外泌体适配体筛选所面临的问题与挑战进行了总结与展望。

## Abstract (en)

Exosomes play crucial intercellular-communication roles and regulate various cellular physiological processes. They are considered potential biomarkers for the early diagnosis of cancers and other diseases. Therefore, detecting and isolating exosomes with specific functions has significant clinical implications. Moreover, the development of low-cost, highly sensitive recognition elements for identifying exosomes is essential for advancing early disease diagnosis and treatment. Nucleic acid aptamers are single-stranded DNA or RNA molecules capable of specifically binding to targets and are produced through the systematic evolution of ligands by exponential enrichment (SELEX) technique. Such aptamers are highly stable, chemically synthesizable, exhibit high affinities and specificities, and are applicable to a broad range of targets, which endow them with unique advantages. Currently, aptamers that target exosomes have been used in a variety of research fields, including cell imaging, drug delivery, and disease diagnosis and treatment. However, selecting aptamers that precisely identify specific exosomes is significantly challenging owing to the complex structures of exosome and their heterogeneity. Consequently, obtaining high-performance aptamers requires efficient screening techniques. This review first summarizes the functions and selection strategies of key targets for exosome-aptamer screening. Furthermore, it outlines the main methods and techniques currently used to screen exosome aptamers, which includes five screening techniques: magnetic bead-SELEX, microfluidic-SELEX, nitrocellulose-SELEX, cell-SELEX, and capillary electrophoresis-SELEX. The separation principles, advantages, limitations, and the latest applications of these techniques are discussed in detail. The review finally addresses current challenges associated with selecting exosome aptamers and provides insight into future research directions.

外泌体是细胞经过“内吞-融合-外排”等一系列调控过程而形成的直径为30~150 nm的圆形单层膜结构的细胞外囊泡^[1,2]^。外泌体含有母细胞的蛋白质、核酸和脂质等重要生物分子,是介导细胞间分子转运及信息交流的多功能载体^[3,4]^。目前,已发现多种类型的细胞在正常及病理状态下均具备分泌外泌体的能力,包括干细胞、免疫细胞、神经细胞以及各类肿瘤细胞等,其外泌体可改变细胞外基质的微环境,将信息传递至受体细胞,并引发病理或生理功能的改变^[5,6]^。此外,外泌体反映了母细胞的表型状态,已成为肿瘤、心血管疾病、神经退行性疾病和免疫疾病的重要生物标志物。外泌体的早期检测不仅能提高患者的生存率,还可避免过度诊断或过度治疗的风险。因此,外泌体的精准识别与鉴定为疾病治疗开辟了新的可能。传统的外泌体检测方法主要包括基于聚合酶链反应(polymerase chain reaction, PCR)的核酸检测、蛋白免疫印迹(Western blot, WB)、酶联免疫吸附测定(enzyme-linked immunosorbent assay, ELISA)、表面等离子体共振(surface plasmon resonance, SPR)、表面增强拉曼散射(surface enhanced Raman scattering, SERS)、荧光检测、微流体技术等^[7⇓⇓⇓⇓⇓-13]^。然而,由于临床样本中外泌体的丰度有限且复杂基质易产生干扰,迫切需要开发灵敏度高、特异性强、易于获取、成本效益高且可靠的检测方法。

基于核酸适配体的生物传感器可实现对外泌体的精准识别与定量分析,成为外泌体检测领域的重要工具。同时,其设计灵活,可适用于多种检测平台,在外泌体检测领域展现出广阔的应用前景。核酸适配体通过指数富集的配体系统进化技术(systematic evolution of ligands by exponential enrichment, SELEX)体外筛选获得,能够与靶标结合的单链脱氧核酸(single-stranded deoxyribonucleic acid, ssDNA)或核糖核酸(ribonucleic acid, RNA)形成G-四链体、发夹、凸环、假结等特殊的三维空间结构,能够以高亲和力与特异性识别结合靶标。与传统抗体相比,作为新型识别分子的核酸适配体具有可化学合成、易于修饰、批次间差异小、低毒性与低免疫原性等独特优势。适配体可通过修饰不同功能的基团,获得信号传感功能,进而构建各种类型的生物传感器,如荧光标记、酶标记、放射性标记等^[14⇓-16]^。其中,基于适配体构建的电化学、荧光和比色传感器等已成功用于肝癌、乳腺癌、胃癌以及前列腺癌等细胞来源外泌体的检测^[17⇓⇓⇓⇓-22]^。

然而,由于外泌体的复杂性及其多样化的生物学特性,筛选出高效、稳定的适配体仍面临诸多技术挑战。目前,在实际应用中,能够满足灵敏度和特异性要求的适配体传感器仍十分有限。高效的筛选技术是获得优良性能适配体的关键。自适配体概念提出^[23]^30多年来,已开发出10多种改良型SELEX技术。目前,适用于外泌体靶标的主要包括固定化和非固定化两大类。基于靶标/寡核苷酸文库固定的技术,通常将完整外泌体或其表面特征蛋白或寡核苷酸文库偶联在固相载体表面,以去除未结合的核酸序列。加入固相载体的优势在于便于将与靶标蛋白结合的序列和未结合序列进行分离,从而提高筛选效率和成功率。然而,固定化可能会改变外泌体特征蛋白的天然构象,进而影响适配体的识别能力。非固定化筛选技术则可以直接在溶液环境中完成筛选。溶液环境能够更好地模拟分子在天然状态下的相互作用,使得筛选条件更接近实际的生理环境,因而可以避免因固定化导致的靶标构象变化,提高适配体性能的稳定性。

目前,针对外泌体的筛选技术主要包括5种(见图1):磁珠-SELEX(magnetic bead-SELEX, MB-SELEX)、微流控-SELEX(microfluidic-SELEX, M-SELEX)、硝酸纤维素膜-SELEX(nitrocellulose-SELEX, NC-SELEX)、细胞-SELEX(Cell-SELEX)和毛细管电泳-SELEX(capillary electrophoresis-SELEX, CE-SELEX)。本文对用于适配体筛选的外泌体特征靶蛋白进行了归纳总结,系统地介绍了外泌体的筛选技术,包括分离原理、技术优势、最新应用以及所面临的问题与挑战,并进一步讨论了高通量测序(next-generation sequencing, NGS)、计算机辅助筛选和化学修饰等新兴技术在外泌体适配体筛选领域的发展趋势及其潜在应用。

**图1:**
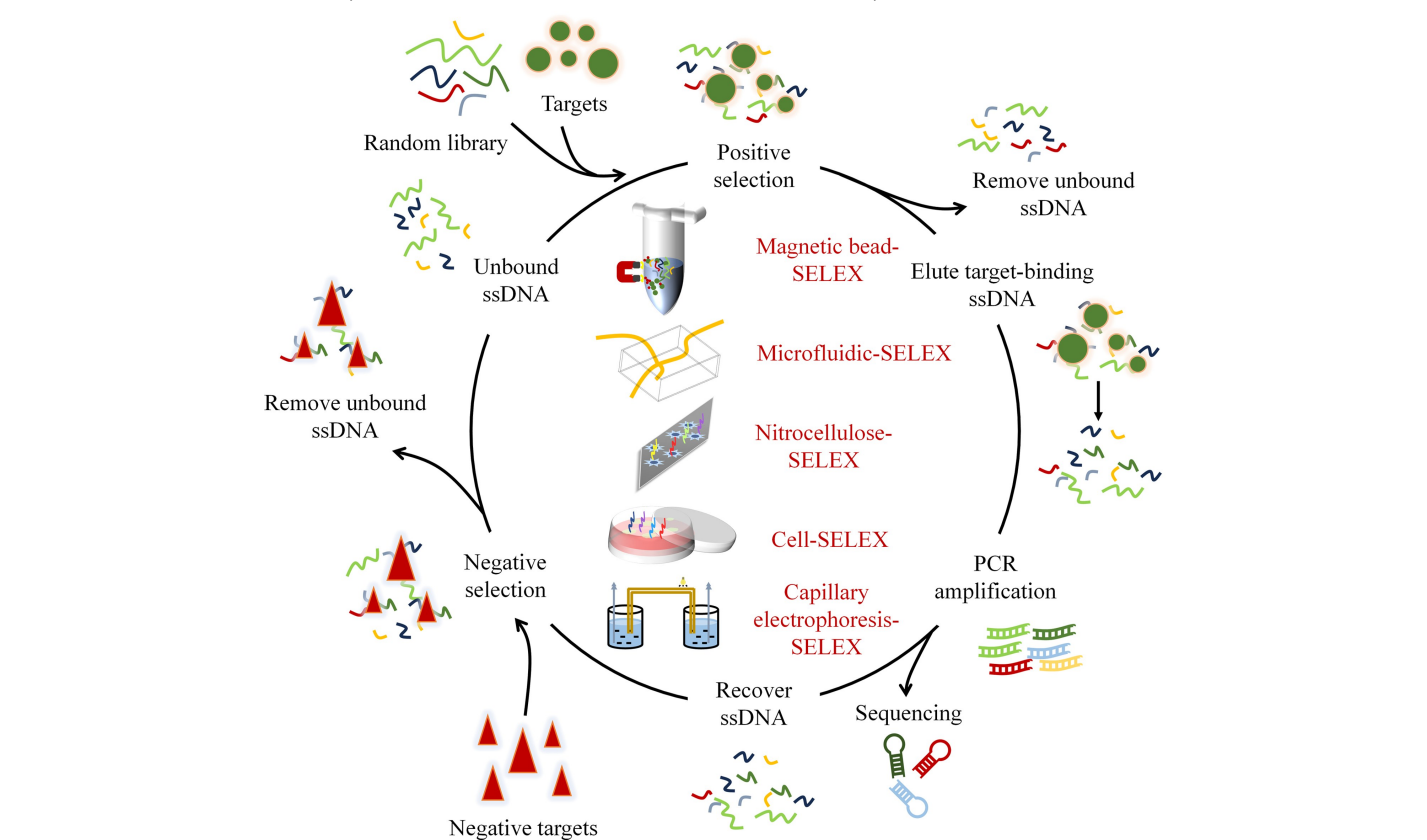
外泌体核酸适配体筛选技术原理

## 1 用于适配体筛选的外泌体特征靶蛋白

大量蛋白质组学研究表明,外泌体的蛋白质组成复杂多样,通常可分为两类,即支持外泌体结构和功能的共有蛋白质以及反映外泌体来源和潜在功能的特异性蛋白质。一方面,共有蛋白质是所有外泌体中普遍存在的蛋白质,主要参与外泌体的形成、分泌、运输和膜结构的维持,支持外泌体的基本生物学功能。这类蛋白质主要包括四跨膜蛋白超家族(CD63、CD81、CD82和CD9)、膜转运和融合蛋白(Annexins和Rabs)、热休克蛋白(Hsp60、Hsp70、Hsc70和Hsp90)等。其中,CD63在外泌体中高度表达,目前已成功筛选得到靶向CD63的适配体,并广泛应用于外泌体的分离与检测。此外,更多的外泌体共有蛋白质被鉴定出来,成为潜在的外泌体适配体筛选的重要靶点。Kalluri团队^[24]^利用定量蛋白质组学发现,Syntenin-1蛋白丰度最高且普遍存在于各种来源外泌体中,揭示其可能成为外泌体的新型生物标志物。Alix和TSG101蛋白是内体分选复合物(endosomal sorting complex required for transport, ESCRT)途径中的重要蛋白质,在不同阶段调控外泌体的形成和释放。其中,Alix蛋白可与Syndecans等膜修饰蛋白相互作用,调节外泌体的产生^[25]^。同样,浮舰蛋白-1(flotillin-1)作为外泌体膜关键结构蛋白,参与囊泡形成和维持外泌体结构完整性^[26]^。目前,针对外泌体共有蛋白质的适配体筛选研究较多,但共有蛋白质在不同来源外泌体中普遍存在,导致以此类蛋白质为靶标筛选得到的适配体难以区分外泌体来源。因此,有必要筛选针对特征蛋白标志物的适配体,实现外泌体来源的鉴定。

特定细胞来源的外泌体具有其特征性的蛋白标志物。例如,树突状细胞和巨噬细胞含有丰富的主要组织相容性复合物体(major histocompatibility complex, MHC),在抗原呈递和T细胞激活过程中发挥着关键作用。而FasL蛋白在自然杀伤(natural killer, NK)细胞来源外泌体中特异性表达。此外,特定肿瘤细胞来源外泌体也包含特异性表达的蛋白标志物。其中,上皮细胞黏附分子(epithelial cell adhesion molecule, EpCAM)在结直肠癌细胞来源外泌体中高表达,糖类抗原125(carbohydrate antigen 125, CA125)是卵巢癌细胞来源外泌体中常见的标志物,人表皮生长因子受体-2(human epidermal growth factor receptor 2, HER2)常见于乳腺癌细胞来源外泌体,而EGFR则与非小细胞肺癌等癌症相关^[27⇓⇓-30]^。目前,已成功筛选出特异性靶向癌症相关外泌体蛋白的适配体,包括EpCAM、HER2、前列腺癌特异性膜抗原(prostate-specific membrane antigen, PSMA)和程序性死亡配体1(programmed death-ligand 1, PD-L1)等。适配体对外泌体特征蛋白的特异性识别,能够在外泌体鉴定和检测中发挥重要作用。

## 2 外泌体的核酸适配体筛选技术

### 2.1 MB-SELEX技术

1996年,Hamm课题组^[31]^首次提出MB-SELEX技术,筛选得到铁蛋白抗体的RNA适配体。MB-SELEX是利用MB作为载体固定寡核苷酸文库或靶标的筛选方法。MB是一种由多层结构组成的磁性颗粒,其核心是通过自由基乳液聚合形成的聚苯乙烯材料。在核心的外部,依次包覆有多层不同的材料,可赋予颗粒特定的化学性质或功能,例如增强磁性或提供反应位点。为满足不同的固定需求,MB表面通常会修饰环氧基、链霉亲和素、羧基、氨基以及甲苯磺酰基等特定的官能团,通过共价结合或生物亲和性将核酸序列或靶标固定在MB表面。在此基础上,通过施加外部磁场除去未结合的核酸序列,而与靶标特异性结合的核酸序列得以保留。此外,MB在水溶液中具有良好的分散性和亲水性,能快速响应外部磁场,目前已成为适配体筛选方法中应用最普遍的固相载体之一。

MB-SELEX的优势在于其较高的富集效率,尤其适用于复杂体系中靶标的筛选。因此,MB-SELEX在外泌体适配体的筛选中具有广泛的应用,能够显著减少非特异性结合,提高适配体的亲和力与特异性,通常经过8~24轮筛选可获得靶向外泌体的适配体^[32,33]^。此外,通过在MB表面修饰干扰蛋白可直接进行负筛,有效排除非特异性结合序列。Niazi等^[34]^首先将MB与牛血清白蛋白(bovine serum albumin, BSA)偶联物作为靶标进行1轮负筛,以去除非特异性结合的序列;在此基础上进一步以乳腺癌细胞来源外泌体中高表达的HER2蛋白为靶标,经过12轮选择获得了靶向HER2蛋白的7条DNA候选序列;其中,适配体H2在序列富集过程中出现频次最高,并且对HER2蛋白具有高亲和力(*K*_D_约为270 nmol/L)。Song等^[35]^以MB作为固相载体,经过12轮筛选获得靶向乳腺癌、胃癌和结肠腺癌细胞来源外泌体中EpCAM蛋白的适配体SYL3。通过截短处理,得到的序列SYL3C(*K*_D_=(38±9) nmol/L, MDA-MB-231; (67±8) nmol/L, Kato Ⅲ)相较于SYL3,亲和力提升了约2~2.5倍。SYL3C可精准靶向Kato Ⅲ和Ramos混合细胞中的EpCAM阳性细胞,从混合细胞中特异性捕获EpCAM阳性的Kato Ⅲ细胞,捕获效率达到63%。因此,适配体SYL3C有望用于EpCAM阳性外泌体的鉴定与分离。

由于MB本身可能会与寡核苷酸文库发生非特异性结合,因此,在MB-SELEX过程中通常会引入MB进行反筛,以提高适配体的特异性。Boyacioglu课题组^[36]^将MB与前列腺癌细胞外泌体表面特征蛋白PSMA偶联筛选10轮后,用不含PSMA蛋白的MB进行2轮反筛,得到适配体SZTI01。并基于DNA适配体二聚体复合物(dimeric DNA aptamer complex, DAC)开发了一种pH敏感型抗肿瘤药物递送系统,将小分子抗癌药物多柔比星(doxorubicin, Dox)靶向递送至PSMA阳性细胞。在酸性环境下,Dox从DAC中释放并进入细胞核,发挥细胞毒性作用,杀伤肿瘤细胞。该系统对PSMA阳性细胞具有高度选择性,而PSMA阴性细胞对DAC的吸收极少,从而降低了药物的系统毒性。而适配体SZTI01对PSMA特异性识别,使其具有被开发成为前列腺癌外泌体适配体传感器的应用潜力。MB-SELEX过程中,以完整的外泌体作为靶标,可增强适配体对天然状态下外泌体的特异性识别能力。Esposito等^[37]^运用“反筛-正筛”交替循环策略,先以MB与正常乳腺上皮细胞来源外泌体的偶联物进行反筛,去除非特异性结合序列,继而使用乳腺癌(breast cancer, BC)细胞来源外泌体进行正筛,富集能够特异性识别BC细胞来源外泌体的序列。经过8轮筛选,最终获得与BC细胞来源外泌体高亲和力结合的适配体ex-50.T(*K*_D_约为0.8 nmol/L),其靶点为HER2蛋白。此外,适配体ex-50.T还可特异性识别BC患者血清中的外泌体。

基于MB-SELEX筛选得到的适配体具有高特异性识别外泌体靶标的优越性能。靶向肿瘤生物标志物的适配体可精准、高效识别与结合肿瘤细胞来源外泌体,促进肿瘤靶向治疗、细胞成像以及捕获新策略的开发。然而,MB-SELEX筛选外泌体适配体存在以下问题与挑战:1)由于需要靶标固定化,完整外泌体作为靶标的固定密度难以控制,高密度的外泌体拥挤可能会阻碍适配体与其相互作用,而密度过低则无法提供足够的结合位点,影响有效序列的富集;2)在固定化过程中,外泌体表面蛋白可能会因与MB等固相载体的结合而导致天然构象发生改变,进而影响适配体的结合位点和筛选结果;3)在MB-外泌体复合物的洗脱过程中,外泌体容易发生破损,导致非特异性结合增加,从而降低筛选效率;4)由于外泌体适配体筛选以及免疫捕获的应用与MB的理化性质密切相关,因此制备理化性质稳定且一致的MB是确保筛选准确性的关键前提;5)外泌体适配体筛选过程中,如何减少MB表面与非外泌体靶标之间的非特异性吸附以提高筛选的准确性和特异性,是亟待解决的问题。

### 2.2 M-SELEX技术

M-SELEX是通过在微流控芯片上固定寡核苷酸文库或靶标的筛选方法^[38]^。利用微加工手段,在硅、玻璃或有机聚合物等材料制成的基质上构建的流体微通道、反应微腔室和储液池等微型结构元件称为微流控芯片,能将样本孵育、分离、检测等过程中的基础功能模块集成在微米级别的芯片中,实现分析过程的全自动化。此外,微流控芯片因其具有集成化、小型化以及样品消耗量小、高通量等特点而备受关注。为实现多样化固定策略,微流控芯片表面通常会修饰特定官能团,如链霉亲和素、氨基、羧基、环氧基以及羟基等,可通过生物亲和性或共价结合等机制将核酸序列或靶标固定在其表面。通过流体冲洗去除未结合的序列,并且使用适当的洗脱缓冲液或通过改变环境条件(如盐浓度或pH值)释放结合的序列。

基于微流控芯片的SELEX技术是一种高效、自动化的适配体筛选方法,可有效实现适配体的快速和高通量筛选,目前已被广泛应用于外泌体适配体的筛选^[39⇓⇓-42]^。MB与微流控芯片结合的筛选技术,即微磁性分离SELEX,可通过微型化的磁力捕获装置实现复合物在微流通道中的分离。由于大部分靶蛋白易偶联至MB表面,因此在芯片微流通道中填充MB介质,可实现靶蛋白-寡核苷酸文库复合物与未结合或弱结合核酸序列的分离。目前,基于微磁性分离SELEX技术已成功应用于多种来源外泌体的适配体筛选,通常经过5~10轮筛选即可得到靶向外泌体的适配体。Huang等^[43]^开发了一种微流控SELEX芯片和竞争性检测芯片相结合的新型微流控系统,实现了孵育、分离和扩增筛选过程的自动化。以肝癌细胞来源外泌体中高表达的甲胎蛋白(alpha fetoprotein, AFP)作为靶标,通过6轮筛选,获得特异性靶向AFP蛋白的适配体,*K*_D_低至2.37 nmol/L。目前,AFP适配体已被应用于肝癌临床诊断的生物传感器开发。Ning等^[44]^利用该适配体构建SERS传感器,可实现肝癌细胞来源外泌体的高灵敏检测。此外,Jin等^[45]^利用荧光标记的AFP适配体与氧化石墨烯(graphene oxide, GO)之间的荧光猝灭效应,并结合酶介导的信号放大策略,成功建立了能高灵敏检测肝癌细胞来源外泌体的方法。

此外,基于MB的M-SELEX技术能在连续的筛选轮次中,直接监测正筛或负筛富集的次级库,分离过程只依赖于外加磁场的作用和微流控系统中的流体流动,无需其他仪器,操作简单方便,成本低,对于外泌体适配体的筛选非常有利。Zhang团队^[46]^开发的多功能微流控筛选平台,同时集成了负筛、正筛区域。首先,在负筛区域利用BSA作为对照蛋白质进行负筛,以去除非特异性结合的序列,在此基础上将未结合的序列引入正筛区域与黏蛋白1(mucin 1, MUC1)结合,富集得到特异性靶向MUC1蛋白的序列。经过2轮“负筛-正筛”循环,最终筛选得到特异性靶向MUC1的适配体T1-20(*K*_D_=(22.4±7.2) nmol/L)。同时,平台通过监测M-SELEX轮次间的荧光强度变化,实现次级库对MUC1(或BSA)亲和力的快速监测。此外,筛选得到的适配体T1-20可成功区分MUC1阳性细胞系(如MCF-7、A549)和阴性细胞系(如T、HaCaT),且T1-20对MCF-7细胞来源外泌体的捕获效率可达到64%。

基于M-SELEX筛选得到的适配体能高效捕获并区分不同来源的外泌体,有望在肿瘤早期诊断以及精准治疗方面发挥重要作用。然而,目前尚无研究使用M-SELEX技术直接利用完整的外泌体进行筛选。此外,在M-SELEX过程中,在固定外泌体特征蛋白靶标时,蛋白可能会因与微流控芯片的结合而导致其天然构象发生改变,进而影响适配体的结合位点和筛选结果。如何减少流体力学效应(剪切力、流速)对适配体与外泌体蛋白靶标结合的影响,也是亟待解决的问题。

### 2.3 NC-SELEX技术

1990年,Tuerk和Gold^[23]^首次提出SELEX技术,并利用NC膜作为分离介质,从含有65536条序列的寡核苷酸文库中成功筛选获得靶向T_4_ DNA聚合酶的RNA适配体。NC膜是一种由纤维素经硝化反应生成的多孔膜过滤材料,因其良好的亲水性、稳定性、高效结合蛋白质的特性而被广泛应用。NC-SELEX利用NC膜作为固相基质,将靶标固定于膜表面筛选结合的核酸序列,并通过加热或使用特定的缓冲液进行回收。由于NC膜具有很强的分子吸附能力,在SELEX过程中能够高效分离与靶标结合的适配体,同时减少未结合序列的干扰。目前,该技术已成功应用于外泌体的DNA适配体筛选,通常经过8~20轮筛选后,适配体的*K*_D_可以达到nmol/L级。Green等^[47]^利用约3×10^14^个ssDNA分子(500 pmol)寡核苷酸文库,以高表达血小板源性生长因子(platelet-derived growth factor, PDGF)的肺癌细胞来源外泌体为靶标,进行12轮筛选,成功获得与PDGF-B链结合的DNA适配体(*K*_D_约为0.1 nmol/L)。该适配体能够区分PDGF的不同亚型并抑制PDGF-BB与其受体α和β结合,阻止细胞增殖,有望成为治疗癌症的PDGF拮抗剂。

通过NC-SELEX可筛选获得识别外泌体特异性表达蛋白的适配体,可精准区分同一标志物的不同亚型。目前,NC-SELEX技术仅用于蛋白靶标的适配体筛选,无法直接以完整外泌体作为靶标。然而,外泌体特异性表达的蛋白质可能会因吸附到NC膜上而发生构象变化,影响适配体的结合方式。此外,NC膜具有较强的非特异性吸附,如何减少NC膜表面与干扰蛋白质之间的吸附,是提高NC-SELEX准确性和特异性的关键。

### 2.4 Cell-SELEX技术

Cell-SELEX是利用完整细胞或外泌体作为靶标的筛选技术^[48]^。Cell-SELEX过程中,细胞或外泌体膜表面蛋白质能维持原有的天然结构和生物活性,有助于确保适配体性能的稳定。Cell-SELEX通常以肿瘤细胞或肿瘤细胞来源外泌体作为靶标,并在第二或第三轮引入负筛步骤,以正常细胞或正常细胞来源外泌体为参照靶标去除非特异性结合序列,实现适配体特异性的进化。Wang等^[49]^采用含有扩展碱基(Z和P)的寡核苷酸文库进行Cell-SELEX,成功获得靶向HepG2细胞来源外泌体的适配体LZH8,其具体靶点为EpCAM, *K*_D_值约为96.0 nmol/L。基于适配体LZH8的强结合特性,将其与DNA纳米四面体结合构建电化学传感器,实现了对HepG2细胞来源外泌体的灵敏检测。Hornung等^[50]^以VCaP细胞来源外泌体为靶标进行1轮正筛,并用LNCaP细胞来源外泌体进行4轮负筛,最终获得一组亲和力在nmol/L级的适配体。其中,适配体Sequence 7能特异性识别VCaP细胞来源外泌体,*K*_D_约为3.0 nmol/L。在此基础上,研究通过生物素标记的Sequence 7与VCaP细胞来源外泌体裂解物共孵育,使用链霉亲和素包被的MB捕获Sequence 7-靶标复合物。进一步加热洗脱与Sequence 7结合的蛋白质,并通过SDS-PAGE进行分离,最终通过液相色谱-质谱鉴定Sequence 7的靶标为Y-盒结合蛋白1(Y-box binding protein 1, YBX1)。

Cell-SELEX技术的优势在于可利用活细胞或完整外泌体进行筛选,能够在复杂体系中识别具有天然构象的靶蛋白。此外,Cell-SELEX技术具有无需靶标纯化、可识别未知靶标等优势,通常经过3~12轮筛选后,适配体的*K*_D_可以达到nmol/L级^[51⇓⇓-54]^。Cell-SELEX筛选获得的适配体能基于膜表面的分子差异而识别不同类型的外泌体,而据此鉴定出的靶标可能成为潜在的肿瘤标志物。然而,Cell-SELEX筛选细胞或外泌体适配体的过程中,仍面临一些问题和挑战,主要包括:1)外泌体膜表面蛋白的数量和种类众多,确定具体的靶标工作量巨大;2)适配体不仅能够与外泌体的膜蛋白选择性结合,甚至会发生内化现象,为适配体的靶标鉴定工作带来挑战;3)Cell-SELEX过程中通常会引入多轮负筛步骤,在提高适配体特异性的同时增加了操作的复杂性,且使筛选时间和经济成本大幅提升。

### 2.5 CE-SELEX技术

2004年,Bowser和Mendonsa^[55]^首次将CE应用于适配体的筛选,建立了CE-SELEX方法。CE-SELEX是以高压电场作为驱动力,并通过毛细管作为分离通道,根据不同分析物在毛细管中的迁移速率差异来实现复合物分离的筛选技术。CE-SELEX过程中无需固定靶标和寡核苷酸文库,二者以天然构象在自由溶液中相互作用,进一步对复合物进行高效分离与自动化的精准收集。此外,CE具有高分辨率、高灵敏度等特性,极大地缩短了筛选周期(1~4轮),是目前公认的高效筛选适配体的方法之一^[56⇓⇓⇓⇓⇓⇓⇓-64]^。CE-SELEX筛选获得的适配体已在医学诊断、环境监测、药物开发等领域得到了广泛应用^[65⇓-67]^。

目前,CE-SELEX已成功用于筛选外泌体靶标的适配体。Zhao等^[68]^提出了一种基于CE-SELEX技术的新型三步进化增强筛选策略,以NK细胞来源的外泌体作为靶标,通过5轮CE筛选,成功筛选得到特异性靶向NK细胞来源外泌体的适配体Apt-6, *K*_D_约为27.6 nmol/L。筛选过程包括3个主要步骤:1)第1轮和第3轮分别以NK细胞来源外泌体的特征蛋白PRF1和FasL(重组蛋白)为靶标,促进适配体候选序列的精确收敛;2)第2轮和第4轮以完整的NK细胞来源外泌体为靶标,提供天然构象的PRFI和FasL蛋白,确保适配体性能稳定;3)第5轮以充间质干细胞来源外泌体为靶标进行负筛,以实现适配体的特异性进化。筛选得到的Apt-6可区分来自不同细胞或体液的外泌体,为从复杂体液中识别疾病相关外泌体提供了更有效的工具。

CE-SELEX的优势在于纳升级的进样量,适用于难获取的生物样本的适配体筛选。同时,无需靶标或寡核苷酸文库的固定化,可进行亚细胞以及细胞结构等复杂结构靶标的适配体筛选。此外,电泳分离过程中可实现复合物的可视化,实时监测适配体序列的富集情况和复合物的生成状态,即时调整筛选策略从而有效提高筛选效率与准确性。基于CE-SELEX筛选得到的适配体具有高特异性,能够识别不同来源的外泌体靶标,可用于肿瘤检测以及靶向治疗。CE-SELEX技术在适配体筛选领域发展较为迅速并趋于成熟,外泌体由于其异质性和结构复杂性,大小、组成、表面标志物之间存在显著差异,因此在CE-SELEX过程中需要引入以纯化后的特征蛋白为靶标的精准收敛步骤,以实现特定外泌体的精准识别。此外,由于外泌体表面含有多种蛋白质,使得其易吸附在毛细管内壁上,导致分离效果下降,可能对筛选效果造成未知影响。

综上,SELEX技术在筛选外泌体适配体中的应用见表1。

**表1:** SELEX技术在筛选外泌体适配体中的应用

Screening strategy	Aptamer		Target		Sequence (5'-3')			Screening round		Affinity *K*_D_/ (nmol/L)	Affinity- characterization method		Ref.
MB-SELEX	EAA (DNA)		CD63		GGGGGTTTGGTTTGGCGGGGTGGCTCCCCGGGGTTG- GTTA			9		51.38±5.12	FA		[32]
	MJ5C (DNA)		PD-L1		TACAGGTTCTGGGGGGTGGGTGGGGAACCTGTT			26		91±12	FA		[33]
	H2 (DNA)		HER2		GGGCCGTCGAACACGAGCATGGTGCGTGGACCTAGG- ATGACCTGAGTACTGTCC			12		270	FA		[34]
	SYL3C (DNA)		EpCAM		CACTACAGAGGTTGCGTCTGTCCCACGTTGTCATGGG- GGGTTGGCCTG			12		22.8±6.0	FA		[35]
	STZI01 (DNA)		PSMA		GCGTTTTCGCTTTTGCGTTTTGGGTCATCTGCTTACGA- TAGCAATGCT			12		-	FA		[36]
	ex-50. T (RNA)		HER2		UGUGGCAGUUAAGAAUAGAUCUUCGCUGCGAUU			8		0.8	ELONA		[37]
M-SELEX	AS2 (DNA)		PSA		GGGCGGGGCGGACGAGACAGTAAGGGCTGTGGGTGT- GGTG			8		0.7	SPR		[42]
	Apt_AFP_ (DNA)		AFP		GGCAGGAAGACAAACAAGCTTGGCGGCGGGAAGGTG- TTTAAATTCCCGGGTCTGCGTGGTCTGTGGTGCTGT			6		2.37	SPR		[43]
	T1-20 (DNA)		MUC1		TCCGAGTTTCCCTGCCCCAACCTCCACCTGGGGTCAA- TAA			2		22.4±7.2	FA		[46]
NC-SELEX		Apt_PDGF_ (DNA)		PDGF		CACAGGCTACGGCACGTAGAGCATCACCATGATCCTG- TGT	12		0.1			NC filter bin- ding method	[47]
Cell-SELEX		LZH8 (DNA)		EpCAM		CATATTAGTACGGCTTAACCCPCATGGTGGACACGGT- GGCTTAGT (P: Artificial nucleotide)	-		96.0			FA	[49]
		Sequence 7		YBX1		CTAGCATGACTGCAGTACGT	5		3.0			ELONA	[50]
		S3 (DNA)		CD109		TAACACGACAGACGTTCGGAGGTCGAACCCTGACAGC- GTGGGC	25		11.93±1.40			FA	[51, 52]
		Sgc8c (DNA)		PTK7		ATCTAACTGCGCGCCGCCGGGAAAATACTGTACGGTT- AGA	20		0.80±0.09			FA	[53]
		LL4A (DNA)		CD63		GCTGGACTCACCTCGACCAGAGCCATTGGGTTTCCTA- GGAAATAGGGCCTTTACTATGAGCGAGCCTGGCG	15		82.18±12.99			FA	[54]
CE-SELEX		Apt-6 (DNA)		PRF1, FasL		TCGGTCGGCTCAGTTGAGGTTTAACCCAGTAGGCGCA- CCA	5		27.6			CE-LIF	[68]

CD63: cluster of differentiation 63; PD-L1: programmed death-ligand 1; HER2: human epidermal growth factor receptor 2; EpCAM: epithelial cell adhesion molecule; PSMA: prostate-specific membrane antigen; PSA: prostate-specific antigen; AFP: alpha fetoprotein; MUC1: mucin 1; PDGF: platelet-derived growth factor; YBX1: Y-box binding protein 1; CD109: cluster of differentiation 109; PTK7: protein tyrosine kinase 7; PRF1: perforin 1; FasL: fas ligand; FA: fluorescence assay; ELONA: enzyme-linked oligonucleotide assay; SPR: surface plasmon resonance; CE-LIF: CE-laser-induced fluorescence.

## 3 总结与展望

核酸适配体作为外泌体研究领域的新兴研究热点,为外泌体的精准识别与靶向递送等应用提供了重要支持。目前,用于外泌体适配体的筛选技术主要包括固定化和非固定化两种。其中,固定化筛选技术操作简便、高效,适合高通量的适配体筛选。而非固定化筛选技术则更有利于保持靶标的天然结构,并可以模拟生物体内环境,获得的适配体具有更大的应用潜力。目前,外泌体的适配体已成为构建生物识别和靶向递送体系的理想工具,能够实现临床样品中外泌体的快速、灵敏检测,并在肿瘤、心血管疾病、神经退行性疾病等早期诊断中展现显著优势。然而,目前可应用于外泌体靶标的适配体数量有限,远少于抗体,且可识别的靶标种类主要局限于少数高表达的特征蛋白,难以满足实际应用需求。因此,提高筛选成功率,获得更多可用的外泌体适配体,是拓展其应用的关键。从生物信息学角度,随着NGS技术的引入,研究者们能够跟踪每轮次级库的序列进化特征和富集趋势,通过生信分析辅助动态调整和优化筛选策略,快速解析适配体库中潜在的高亲和力序列,从而缩短筛选周期并提高成功率。此外,计算机辅助技术是目前用于优化、改造适配体序列和结构的主要手段。构建外泌体适配体的计算机辅助筛选平台,以实现外泌体适配体的虚拟筛选、结构预测与优化以及分子动力学模拟研究,将为适配体的结构设计、优化以及结合机制提供重要指导。此外,由于外泌体是纳米级别的囊泡、密度低且易在生物体液中分散,因此适配体在循环体液中识别外泌体时,需具备长时间循环和抗降解等特性,确保拥有更高的稳定性和有效性。在筛选过程中,通过引入聚乙二醇(polyethylene glycol, PEG)、甲基化等化学修饰,利用锁核酸(locked nucleic acid, LNA)和肽核酸(peptide nucleic acid, PNA)等人工寡核苷酸文库,能够显著提升适配体在体内的抗降解能力和循环时间。同时,也应关注适配体修饰对其结合能力与细胞毒性的影响。NGS、计算机辅助筛选和化学修饰以及人工核酸等多种新兴技术的迅速崛起,也为外泌体核酸适配体的高效筛选和结构改造提供了新策略和新视角,推动外泌体核酸适配体筛选技术更趋完善。

## 作者团队简介

北京理工大学生命学院屈锋教授课题组自2005年以来深耕于多模式毛细管电泳在大健康领域的分析研究和应用、毛细管电泳分析仪器研制、毛细管电泳-质谱联用技术等研究方向, 在毛细管电泳基础和应用研究、核酸适配体筛选机理及应用研究领域形成了研究特色。已承担多项国家级科研项目, 并与国内外相关领域的知名学者建立了广泛的合作关系。

**Table:** 

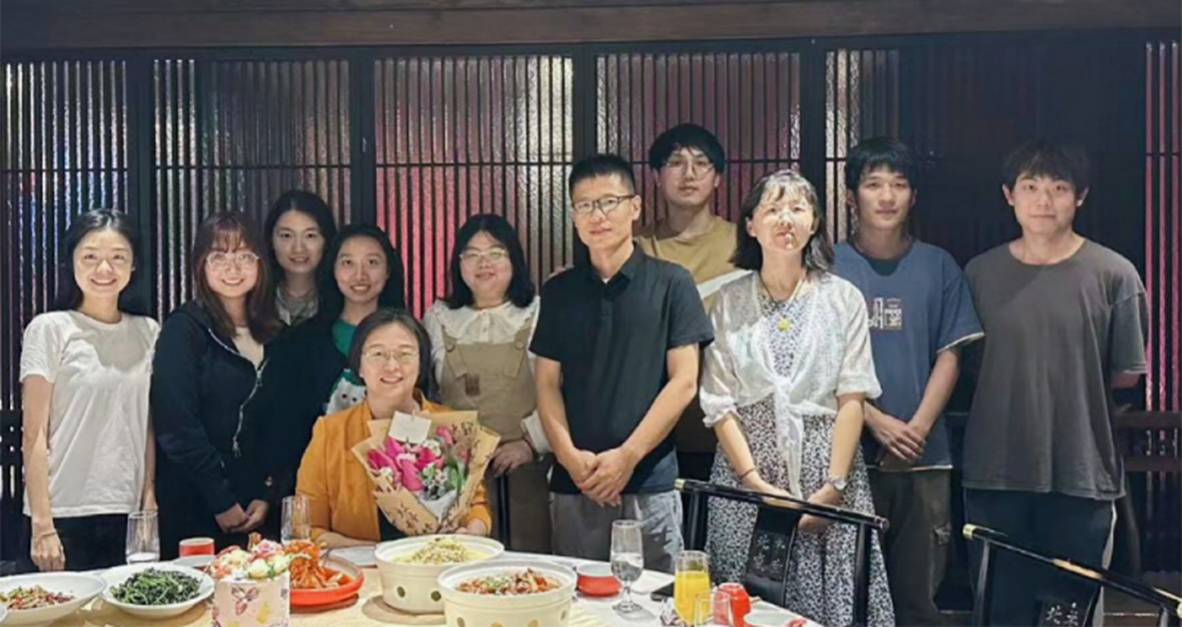	人才队伍
	**学术带头人:**屈锋教授 **在读学生:**研究生5人 **团队精神:**锲而不舍,金石可镂

**Table:** 

科研项目及成果
**科研项目:**国家自然科学基金,北京市自然科学基金 **科研成果:**在*Signal Transduction and Targeted Therapy*、*Biosensors and Bioelectronics*、*Sensors and Actuators B*: *Chemical*、*Chinese Chemical Letters*、*Analytical Chemistry*、*Talanta*等期刊发表科研论文100余篇,他引1600余次,授权发明专利9项,申请专利2项。 **获奖情况:**中国分析测试协会科学技术奖CAIA奖一等奖
研究领域
**研究方向:** (1)生物医学分析检测;(2)生物分离 **研究内容: **(1)蛋白质与核酸分析及相互作用研究;(2)蛋白质组学分析方法研究;(3)生物分离新材料;(4)细胞活性分析及毒性物质评价;(5)微生物特性及分析检测研究
